# Influence of cryopreservation on the antioxidative activity of *in vitro* cultivated *Hypericum* species

**DOI:** 10.1080/13102818.2014.946805

**Published:** 2014-11-07

**Authors:** Elena Georgieva, Detelina Petrova, Zhenya Yordanova, Veneta Kapchina-Toteva, Eva Cellarova, Ganka Chaneva

**Affiliations:** ^a^ Faculty of Biology, Sofia University ‘St. Kliment Ohridski’, Sofia, Bulgaria; ^b^ Faculty of Science, Institute of Biology and Ecology, Pavol Jozef Šafárik University of Košice, Košice, Slovak Republic

**Keywords:** *Hypericum* rumeliacum, *Hypericum* tetrapterum, cryopreservation, phenols, flavonoids

## Abstract

Antioxidative activity of two *in vitro* cultivated *Hypericum* species – *H. rumeliacum* Boiss. and *H. tetrapterum* Fr. – was estimated after cryopreservation. Both species were successfully regenerated after a cryopreservation procedure performed by the vitrification method. *H. tetrapterum* did not manifest any significant oxidative stress-induced changes caused by low-temperature treatment. Conversely, a decrease in green pigments' content of *H. rumeliacum* was measured, particularly pronounced in chlorophyll *b*, which was accompanied by an increase of carotenoids in the regenerated plants.

A strong increase of malone dialdehyde and H_2_O_2_ levels in *H. rumeliacum* tissues was detected. Superoxide dismutase activity was enhanced by 170%, as well as the catalase activity, which was 220% above the control. The same trend was observed in *H. tetrapterum*, although less pronounced – 143% increase of superoxide dismutase and 112% of catalase.

Cryopreservation did not influence the phenol content in the examined plants, but it led to an increase of flavonoid content, especially in *H. tetrapterum*, by 237%. Total antioxidant activity in regenerated *H. tetrapterum* varied around the control level, but it was increased in *H. rumeliacum*. The free proline content in *H. tetrapterum* remained almost unaffected after freezing, as opposed to *H. rumeliacum*, where a strong increase of proline content (208% above the control) occurred. An electrolyte leakage from the cells of *H. rumeliacum* regenerated after cryopreservation was also registered, albeit not significant.

## Introduction

Plant species of the genus *Hypericum* are among the most important medicinal plants.[1,2] *Hypericum rumeliacum* is a rare species, characteristic for the Balkan flora, with conservational importance for Bulgaria. A number of studies report on its phytochemical composition and valuable pharmacological properties.[3–5] In this regard, the development and improvement of protocols for cryopreservation and *in vitro* cultivation are one of the most important tasks designed to preserve and propagate these valuable and often endangered species, such as members of genus *Hypericum*.

Among various environmental stresses, low temperature is one of the most important factors limiting the productivity and distribution of plants and furthermore, it is also responsible for the production of ROS (reactive oxygen species) in the plant cell.[6,7] It is established that cryopreservation also causes ROS-mediated oxidative stress, which could occur in every step of cryopreservation protocol associated with dehydration.[8,9] Low-temperature-induced oxidative stress increases lipid peroxidation, and alters the enzyme activities and plant physiological processes.[10] At a physiological level, photosynthesis is strongly affected by exposure to cold [11] and photosynthetic pigments are highly sensitive to the deleterious effect of low temperatures. Freezing destroys chlorophyll biosynthesis, inhibits thylakoid electron transport and reduces photosynthesis.[12] Low temperature causes a significant reduction in chlorophyll *a* and chlorophyll *b*, as well as significant changes in carotenoid composition.[13]

Low temperatures affect substantially enzyme activity in plants.[14] The activity of some antioxidant enzymes is partially correlated with the plant sensitivity to cold and, thus, the antioxidant enzymes possess a significant importance in the chilling tolerance.[15] It was found that the extreme low temperatures could induce an increase of SOD (superoxide dismutase) activity and a decrease in CAT (catalase) activity.[16]

High proline accumulation, in the plant cells under stress conditions, could significantly increase the capacity for ROS detoxifying and for lowering the oxidative damage. The similar effect could be observed in exogenous proline treated cells (as a part of the cryopreservation protocol). There are many studies where cellular membranes have been shown as the primary site of freezing injury in plants.[17–20] The increased membrane permeability enhances ion leakage, allows the entrance of undesirable ions into the cell and hampers the osmosis and diffusion, etc.

According to our previous results, *in vitro* cultivated *H. rumeliacum* gave relatively low rates of plant survival but it is important to note that it produced high levels of phenolic and flavonoid compounds comparable to those in intact plants.[21] It is well established that the phenolic compounds are mainly responsible for the unique medicinal characteristics of the *Hypericum* species and they are among the main reasons for the enhanced scientific interest in recent years.[1,2,5] As we have previously observed, the levels of total polyphenolic and flavonoid compounds are commensurable for both species – *H. rumeliacum* and *H. tetrapterum* – but total hypericin is much higher in *H. rumeliacum*.[22] Since phenolic and flаvonoid compounds were in commenrurable levels for these two species, the high levels of total hypericins in *H. rumeliacum in vitro* made this species a very intriguing object for further investigation. This gave us a reason to continue the research on both experimental plants and their metabolic activities.

The aim of the present work was to evaluate the antioxidative status of *H. rumeliacum* and *H. tetrapterum* regenerated after cryopreservation.

## Material and methods

The experimental plants, *H. rumeliacum* Boiss. and *H. tetrapterum* Fr. (voucher specimen deposited at the Herbaruim of the Institute of Botany, Bulgarian Academy of Sciences, Sofia-SOM 163 524), were cultivated on MS (Murashige and Skoog) medium at a temperature of 25 °C, 16/8 h photoperiod and light intensity of 0.000060 mol m^−2^ s^−1^ with a 45-day period of regular subculture. Cryopreservation was performed by the method of vitrification; shoot tips were pre-cultivated with 0.076 μmol/L АВА (abscisic acid) for 10 days and afterwards equilibrated for 90 min in the ice at 0 °С. Furthermore, the regeneration was performed on the MS medium containing 0.5 mg l^−1^ BA (benzyladenine).

Chlorophyll and carotenoid contents were determined after 80% acetone extraction according to Arnon et al.[23] Endogenous hydrogen peroxide (H_2_O_2_) was determined spectrophotometrically at 390 nm after the reaction of the plant extract with 1 mol/L KI. Values were calculated using a standard curve.[24] Malone dialdehyde (MDA) content was measured according to Dhindsa et al.[25] Concentration of MDA was calculated by means of an extinction coefficient of 15,5000 L mol^−1^ cm^−1^. SOD activity was measured after Beauchamp and Fridovich.[26] CAT activity was determined according to Aebi.[27] Total phenolic content was determined according to Singleton et al.,[28] total flavonoid content – according to Chang et al.[29] and total antioxidant activity – according to Prieto et al.[30] Proline content was measured as it was described by Bates et al.[31]

Leaf membrane damage was estimated by recording the electrolyte leakage. Plant material was washed with deionized water and placed in tubes with 30 ml of deionized water and incubated for 24 h at 25 °C in a dark environment. The electrical conductivity of the solution was measured by HI 255 Combined Meter (Hanna Instruments). Samples were autoclaved at 1 atm for 30 min and the conductivity was measured again. The results were presented as % (it was accepted that the conductivity after autoclaving was 100%).

All experiments were repeated three times under the same conditions. The data were averaged of triplicate measurements. Тhe significance of differences between control and each treatment was determined using Student's *t*-test, *p* ≤ 0.05.

## Results and discussion

In general, *H. rumeliacum* and *H. tetrapterum* were successfully regenerated after cryopreservation. We did not observe any visible differences between control and regenerated *H. tetrapterum* plants. A higher tolerance for low temperature was demonstrated, while regenerated *H. rumeliacum* plants showed slower growth rates, shorter stems, fewer leaves and poorly developed root system.

Photosynthetic pigments in *H. tetrapterum* did not change significantly after cryopreservation which confirmed the notion for that species as more tolerant to a freezing procedure (Table 1). A considerable decrease of green pigments’ content in *H. rumeliacum* (especially of chlorophyll *b*) was measured, accompanied with an increase of carotenoids. An 18% decrease of chlorophyll *a* in *H. rumeliacum* was measured after regeneration. Chlorophyll *b* was more significantly influenced by low temperature – its content was 26% decreased in *H. rumeliacum* tissues and 8% decreased in *H. tetrapterum* (Table 1). Similar results were obtained by Rahnavard et al.[32] who observed that the higher altitude in the mountains combined with low temperature, provoked reduced chlorophyll biosynthesis in the low-tolerant *Hypericum* species, especially in *H. rumeliacum*.

**Table 1.** Influence of cryopreservation on the content of plastid pigments (mg/g FW, % of control) in the tissues of *H. rumeliacum* and *H. tetrapterum*.

	Chlorophyll *a*		Chlorophyll *b*		Carotenoids	
Variants	(mg/g FW)	(% of control)	(mg/g FW)	(% of control)	(mg/g FW)	(% of control)
*H. rumеliacum* control	0.77 ± 0.03	100	0.43 ± 0.01	100	0.13 ± 0.006	100
*H. rumeliacum* cryo	0.63 ± 0.02	82	0.32 ± 0.01	74	0.17 ± 0.007	131
*H. tetrapterum* control	0.88 ± 0.03	100	0.51 ± 0.02	100	0.19 ± 0.007	100
*H. tetrapterum* cryo	0.90 ± 0.04	102	0.47 ± 0.02	92	0.20 ± 0.007	105

Carotenoid content of *H. tetrapterum* was only slightly changed (5% increased) after cryopreservation. On the contrary, carotenoids in *H. rumeliacum* were 31% increased, which indicated a possible protective role against oxidative stress (Table 1). It is well documented that carotenoids may act as antioxidants, which functions include membrane protection against free radicals’ damage and their abundance increases at low temperatures.[33,34] It has been demonstrated that growth at low temperature considerably modifies the pigment composition and the large reduction in contents of chlorophylls accompanied by the accumulation of large amounts of the de-epoxidized xanthophylls occurred.[35]

Membranes are a primary site of cold-induced injury. It was previously found that there is a slight increase of MDA and Н_2_О_2_ levels after cryopreservation,[21,36] which may be indicative for the overcoming of oxidative stress and the recovery of the physiological status of regenerated *H. rumeliacum*. An increased accumulation of MDA and ROS occurred in regenerated *Hypericum* plants after cryopreservation, but the biosynthetic capacity of the regenerated plants was not impaired by the freezing procedure. Moreover, the cryopreserved plants showed higher phenolic and flavonoid contents and possess increased antioxidant capacity than those of the unfrozen controls.[9]

After cryopreservation, both experimental species showed enhanced values of MDA and H_2_O_2_, most considerable in *H. rumeliacum* (Table 2). Insignificant levels of MDA in the tissues of *H. tetrapterum* in the control and regenerated plants were observed. On the contrary, MDA content in the regenerated *H. rumeliacum* was two times higher than that in the control plants, which could be regarded as evidence of increased lipid peroxidation caused by the cryopreservation.

**Table 2.** Influence of cryopreservation on the malone dialdehyde (MDA) content (mM/g FW, % of control) and Н_2_О_2_ content (μM/g FW, % of control) in *H. rumeliacum* and *H. tetrapterum*.

	MDA		H_2_O_2_	
Variants	(mM/g FW)	(% of control)	(μM/g FW)	(% of control)
*H. rumeliacum* control	0.21 ± 0.008	100	5.05 ± 0.16	100
*H. rumeliacum* cryo	0.52 ± 0. 024	250	7.88 ± 0.32	156
*H. tetrapterum* control	0.015 ± 0.0006	100	1.95 ± 0.081	100
*H. tetrapterum* cryo	0.018 ± 0.0008	120	2.18 ± 0.076	112

It is well known that Н_2_О_2_ overproduction induced by ROS and other stress conditions leads to disturbance in plant metabolism. We found that H_2_O_2_ content was affected less in comparison with MDA (Table 2). The levels of hydrogen peroxide in cryopreserved *H. rumeliacum* plants were highly increased (156% compared to the control). In the tissues of *H. tetrapterum* that increase was less pronounced – 12% above the control. These results confirmed the view of *H. rumeliacum* as a more sensitive species to the cryopreservation protocol that was used.

The reduced activity of antioxidant enzymes in extreme cold is a common effect which accelerates the accumulation of ROS in higher amount. Yang et al.[37] have found that chilling stress reduced the activities of antioxidant enzymes viz. SOD, POD, CAT and APX in *Cucumis sativus*. Meanwhile, it was shown[37,38] that the enhanced activities of SOD, CAT, APX and POX in some plants reflected in a better tolerance to chilling. It has been also found [39] that there is an increased CAT and peroxidize activity in *Hypericum* plants growing at a higher altitude. Thus, activated protein synthesis and increased antioxidative enzymes’ activity play a significant role in the protection against stress conditions in the high mountains.

We observed that cryopreservation induced an increase in SOD and CAT activities in both experimental species. According to our results, the activity of SOD in *H. rumeliacum* tissues was highly increased – 170% above the control variant. That increase was less expressed in *H. tetrapterum* plants – 143% as compared to the control (Table 3). That rise in the activity of SOD could be considered as a consequence of the enhanced content of its substrate – superoxide anion.

**Table 3.** Changes of superoxide dismutase (SOD) activity (U mg^−1^ prot., % of control) and catalase (CAT) activity (Δ*E* min^−1^ mg^−1^ prot, % of control) in *H. rumeliacum* and *H. tetrapterum* after cryopreservation.

	SOD		CAT	
Variants	(U mg^−1^ prot.)	(% of control)	(Δ*E* min^−1^ mg^−1^ prot)	(% of control)
*H. rumeliacum* control	76.63 ± 33.60	100	5.57 ± 0.22	100
*H. rumeliacum* cryo	130.27 ± 5.08	170	12.25 ± 0.44	220
*H. tetrapterum* control	48.07 ± 2.40	100	14.24 ± 0.66	100
*H. tetrapterum* cryo	68.74 ± 3.29	143	16.38 ± 0.62	115

CAT activity in *H. rumeliacum* cells showed a strong increase after cryopreservation (220%), while the enzyme activity was only slightly increased in *H. tetrapterum* plants regenerated after cryopreservation (112%), (Table 3). The significant increase of CAT activity in cryopreserved *H. rumeliacum* plants could be used as evidence for ROS formation and development of oxidative stress. In the scientific literature there are many controversial publications about the effects of low temperature on CAT activity. CAT is generally considered to be more labile enzyme at low temperatures as compared to SOD. It is possible that the enzyme is damaged by OH**^.^** or by the excess of excitation energy which is not transformed via photosynthesis and further initiates photo-oxidative damage which in turn, affected negatively CAT.[40,41] It has been shown, however, that low-temperature treatments led to a significant increase in total CAT activity and resulted in isoenzyme pattern shifts.[42] It was therefore not surprising for us to measure the increase in the activity of CAT in *Hypericum*.

It is considered that natural antioxidants with their multifunctional activities may serve as a good alternative of synthetic compounds in preventing the oxidative damage.[43] Those activities depend on a number of parameters including the experimental conditions. It was found that the broad profile of phenolic compounds determine their key role in detoxifying of free radicals in *Hypericum* species.[44] Rahnavard et al.[32] observed *Hypericum* species growing at the highest altitude and lowest temperature respectively, contain the highest quantity of hypericin and total phenolic content. On the contrary, the highest flavonoid content was measured in the ecotypes growing at the lowest altitude.

**Figure 1.**
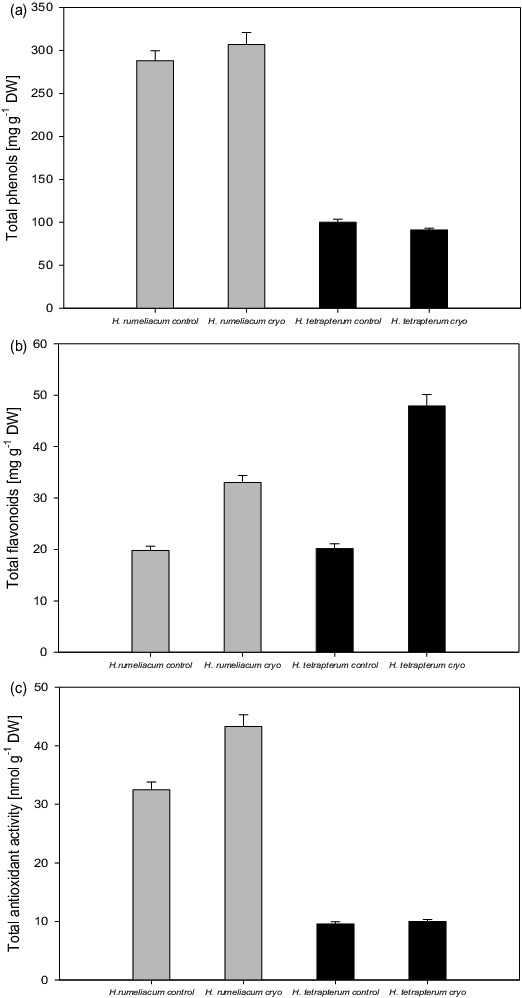
Influence of cryopreservation on the secondary metabolites’ accumulation in the tissues of *H. rumeliacum* and *H. tetrapterum*; A – changes in total phenolic content; B – changes in total flavonoid content; C – changes in total antioxidant activity.

Cryopreservation did not affect negatively the phenolic and flavonoid biosynthesis in both experimental plants. Phenolic biosynthesis was not influenced by the cryopreservation procedure in both experimental plants (Figure 1(A)) and the values varied around the control levels. It is important to emphasize that *H. rumeliacum* showed significantly higher biochemical capacity for synthesis of phenolic compounds as compared to *H. tetrapterum*.

Flavonoids accumulate in leaves and stems in response to low temperatures. Recently, it has been reported that cold stress increases flavonoid biosynthesis.[45] Besides, part of the valuable properties of *Hypericum* species was attributed mostly to flavonoid compounds. Both experimental species showed an increased flavonoid biosynthesis which is an unexpected beneficial effect caused by cryopreservation (Figure 1(B)). Flavonoid content was particularly increased in *H. tetrapterum* tissues – 237% as compared to the control. It was observed that total antioxidant activity in the cryopreserved *H. tetrapterum* varied around control values while in the regenerated *H. rumeliacum* plants that parameter was increased (33% above the control) and that indicated the presence of oxidative stress and lower tolerance towards cryopreservation (Figure 1(C)).

In response to cold and other osmotic stresses, plants accumulate a range of compatible solutes including proline.[14,46] It is well known that accumulation of proteins, carbohydrates and proline plays a major role in the plants’ survival at high-altitude conditions.[39] Proline is successfully applied in the process of cryopreservation of plant cells due to its osmo-protective properties, as well as its importance as a regulator of cellular ROS balance.[47]

**Figure 2.**
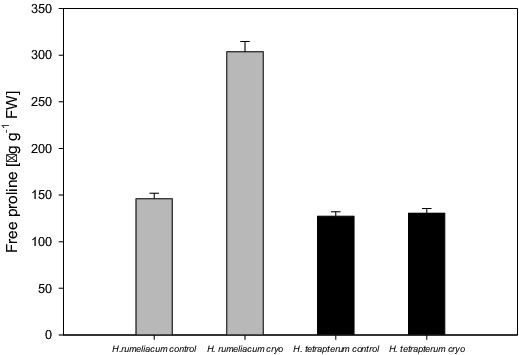
Changes of free proline content in *H. rumeliacum* and *H. tetrapterum* under cryopreservation.

Proline content did not increase in the regenerated *H. tetrapterum* plants (Figure 2). In the regenerated *H. rumeliacum* the concentration of free proline was increased twice compared to control values, which could be a clear indication for the presence of oxidative stress and low tolerance of that species to freezing.

**Figure 3.**
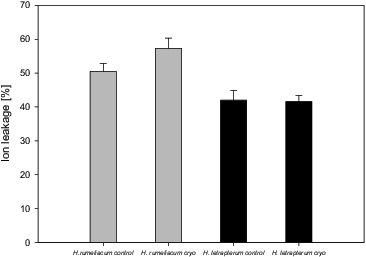
Changes of ion leakage from *H. rumeliacum* and *H. tetrapterum* tissues, caused by cryopreservation.

Ion leakage could be used as an indicator for the level of membranes’ injuries caused by different stress factors. As the low temperature disrupts the integrity of the cell membranes, it is possible that the inhibition of membrane associated reactions under low temperatures is attributed to increased membrane permeability.[19] We observed an enhanced ion leakage (57.3%) from the cells of *H. rumeliacum*, which was an acceptable reason to assume that some membrane damages took place as a consequence of the processes of dehydration and freezing occurring during cryopreservation. No significant differences between the control and regenerated *H. tetrapterum* plants (Figure 3) were found. These results were in agreement with changes that were measured in MDA, H_2_O_2_ and the carotenoid content (Tables 1 and 2) and, thus, the idea that membranes of cryopreserved *H. rumeliacum* plants were affected in some extent by low temperature (increased ion leakage) was confirmed.

## Conclusion

The results that were obtained gave us a good reason to suggest that *H. tetrapterum* is highly tolerant towards low temperature and cryopreservation. No disturbances in growth, biochemical and cellular processes were registered, nor were there any indications of oxidative stress. On the contrary, *H. rumeliacum* was less tolerant to cryopreservation. It showed a reduced pigment synthesis, high levels of MDA and H_2_O_2_ in the tissues, increased activities of SOD and CAT and some evidence for oxidative damage of the cell membranes. Taking into account that *H. rumeliacum* is an endemic species, very rich in hypericin, it is necessary to optimize the cryopreservation protocol aiming to reduce the oxidative stress symptoms in the regenerated plants and, thus, to ensure better survival and conservation.
